# Co-optimization method to improve lateral resolution in photoacoustic computed tomography

**DOI:** 10.1364/BOE.469744

**Published:** 2022-08-09

**Authors:** Yang Zhang, Shufan Yang, Zhiying Xia, Ruijie Hou, Bin Xu, Lianping Hou, John H. Marsh, Jamie Jiangmin Hou, Seyed Mojtaba Rezaei Sani, Xuefeng Liu, Jichuan Xiong

**Affiliations:** 1 School of Electronic and Optical Engineering, Nanjing University of Science and Technology, Nanjing 210094, China; 2 School of Computing, Edinburgh Napier University, Edinburgh, Scotland, EH10 5DT, UK; 3 James Watt School of Engineering, University of Glasgow, Glasgow G12 8QQ, UK; 4 The Royal College of Surgeons of Edinburgh, Nicolson Street, Edinburgh, Scotland, EH8 9DW, UK; 5 Department of Physics, North Tehran Branch, Islamic Azad University, Tehran 16511-53311, Iran; 6 Equal Contribution; 7 liuxf_1956@sina.com

## Abstract

In biomedical imaging, photoacoustic computed tomography (PACT) has recently gained increased interest as this imaging technique has good optical contrast and depth of acoustic penetration. However, a spinning blur will be introduced during the image reconstruction process due to the limited size of the ultrasonic transducers (UT) and a discontinuous measurement process. In this study, a damping UT and adaptive back-projection co-optimization (CODA) method is developed to improve the lateral spatial resolution of PACT. In our PACT system, a damping aperture UT controls the size of the receiving area, which suppresses image blur at the signal acquisition stage. Then, an innovative adaptive back-projection algorithm is developed, which corrects the undesirable artifacts. The proposed method was evaluated using agar phantom and ex-vivo experiments. The results show that the CODA method can effectively compensate for the spinning blur and eliminate unwanted artifacts in PACT. The proposed method can significantly improve the lateral spatial resolution and image quality of reconstructed images, making it more appealing for wider clinical applications of PACT as a novel, cost-effective modality.

## 1. Introduction

**Fig. 1.**
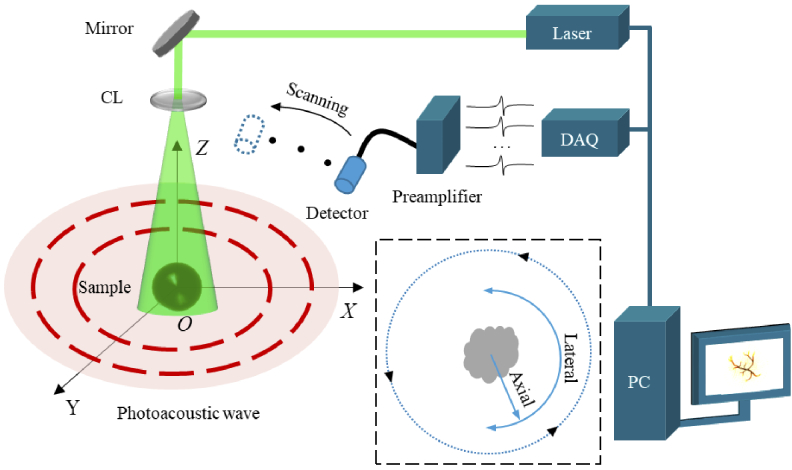
The photoacoustic computed tomography system. The red dashed circle represents the photoacoustic wave. The dotted inset shows the scanning geometry and the directions of axial and lateral resolution. CL, concave lens; DAQ, data acquisition system; PC, personal computer.

Photoacoustic Computed tomography (PACT), also known as optoacoustic tomography, is a novel technique of non-invasive medical imaging based on the photoacoustic effect [1,2]. In PACT, a sufficiently short-pulsed laser is employed to irradiate a target sample such as biological tissue. The resulting photoacoustic effect generates photoacoustic (PA) waves within the sample. After propagating away from the sample, these signals are collected by wideband ultrasonic transducers (UTs) [3,4], as illustrated in Fig. 1. The goal of PACT is to reconstruct an image from the acquired signals which represents the distribution of optical absorption within biological tissue by using a reconstruction algorithm [5–7]. PACT combines the advantages of excellent optical absorption contrast and depth of acoustic penetration depth within tissues. It has been an essential tool in many active areas of biomedical imaging [8–10].

In PACT systems, the scanning method and the reconstruction algorithm directly affect the quality of the reconstructed image. Three commonly used scanning geometries are planar, cylindrical, and circular/spherical surfaces [11]. Compared with the other two geometric scanning methods, circular scanning is widely used because signals are collected over a broad range of angles and it is low cost. In circular scanning PACT, the axial resolution is spatially invariant and is limited by the bandwidth of the UT, while the lateral resolution is spatially variable and is largely determined by the element size of the UT [12,13]. For a UT with a large aperture, the lateral resolution of images is poor when far away from the scanning center compared with images near the scanning center [14]. Meanwhile, among all the current reconstruction algorithms, the back-projection (BP) algorithm is widely used due to its low computational complexity and memory demand. The BP algorithm assumes that the UT is an ideal point detector. However, the mismatch between the real-life finite UT element size and the point detector estimation in the algorithm causes undesirable artifacts in the reconstructed image [15]. Two common artifacts are negative artifacts with strong negative optical-absorption values at the edge of the image [16,17] and spinning blurring artifacts in the lateral direction of the image [18]. The axial and lateral resolutions are defined within the dashed box inset in Fig. 1, the axial resolution is perpendicular to the scanning direction, while the lateral resolution is parallel to the scanning direction.

To overcome the above difficulties and improve the quality of the reconstructed image, the most obvious solution is to use a UT with a small aperture or to move the detector away from the imaging area (that is, increase the scanning radius). However, it is not practically feasible to increase the scanning radius due to space constraints. In addition, a UT with a small aperture has a high cost (with an aperture diameter of 5 mm costing at least $1500 ) and low sensitivity. The problem of finding a balance between the UT aperture and its sensitivity has not yet been resolved. A virtual point detector with a large numerical aperture [19,20] or attaching an acoustic concave lens in front of the detector [21,22] could improve the lateral resolution and reduce the sensitivity of the detector; however, it is more expensive to customize the detector with built-in accessories.

Attempts have also been made to modify model-based reconstruction algorithms to improve the lateral resolution [23,24]. These methods can enhance the image quality by introducing constraints to suppress image artifacts but at the cost of large matrix calculations and increased demand for computer memory. Alternatively, the recent success of deep learning approaches in solving complex imaging problems has seen an increasing interest in applying similar strategies to improve the lateral resolution of PACT [25,26]. However, methods based on deep learning require vast quantities of data to train the network, which demands considerable computing power and expensive hardware.

In this paper, a damping aperture UT and adaptive back-projection co-optimization (CODA) method is developed to improve the lateral resolution. We first report the design of a damped UT to improve the lateral resolution by controlling the size of the receiving area. Then, an adaptive back-projection reconstruction algorithm is developed, which corrects undesirable image artifacts. Improvements in image quality can be observed all in simulation, agar phantom and *ex-vivo* chicken heart experiments.

## 2. Methods

### 2.1 Modified ultrasonic transducer approach

**Fig. 2.**
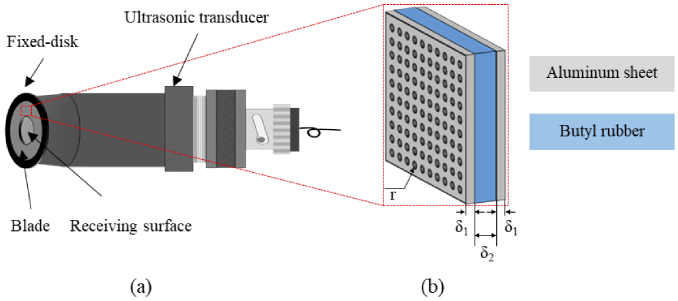
Schematic diagram of the damped ultrasonic transducer: (a) external view, (b) structure of the composite blade. r is the radius of the small holes on the aluminum sheet, which is 0.6 mm. The distance between rows and columns of small holes is 0.4 mm. The thickness of aluminum sheet δ_1_ and butyl rubber δ_2_ is 0.2 mm and 1 mm, respectively.

An aperture damper composed of a fixed disk and blade was installed on the receiving surface of the UT. Here, an unfocused UT (Doppler Inc., element diameter: 10 mm) was used, with a center frequency of 5 MHz and a bandwidth of 79% at -6 dB, which costs around $350 . This configuration is illustrated in Fig. 2(a).

The blade consisted of two thin aluminum sheets bonded together with a sheet of butyl rubber in between them, which has the effect of blocking the transmission of ultrasound waves. The numerous perforated holes in the aluminum sheet increase the absorption of ultrasound, and also reduce the impact of vibrations that are generated when the sound wave propagates to the surface of the aluminum sheet, as shown in Fig. 2 (b). The central aperture size of the blade can be trimmed. The blade and the fixed disc are connected by waterproof glue. For comparison, three aperture sizes with diameters of 10 mm (equivalent to the traditional UT), 6 mm and 2 mm are tested respectively. Essentially, the receiving angle range of the UT is controlled by changing the size of the central aperture of the blade. In this way, the receiving angle of the detector can be increased at a low cost without reducing the area of the internal piezoelectric crystal, maintaining sufficient sensitivity to ensure the accuracy of the reconstructed image.

### 2.2 Adaptive back-projection algorithm

The most well-known reconstruction technique for PACT is the back projection (BP) algorithm [11] which is mathematically based on the direct inversion of the circular or spherical Radon transform [17]. The formula for the BP algorithm can be written as

(1) $$p_{0}(r)=\frac{1}{\Omega_{0}}\underset{\text{S}}{\int }\text{d}\Omega_{0}{\left[2p_{d}(r_{0},t)-2t\frac{\partial p_{d}(r_{0},t)}{\partial t}\right]|}_{t=|r_{0}-r|/c_{0}}.$$

where $c_{0}$ is the speed of sound; $p_{d}$ is the detected signal; $\Omega_{0}$ is the solid angle subtended by the total detection surface *S* and $d\Omega_{0}$ is the solid angle subtended by the total detection surface *S* with respect to a source point at *r*. The term $d\Omega_{0}/\Omega_{0}$ is a factor weighting the contribution to the reconstruction from the detection element dS .

**Fig. 3.**
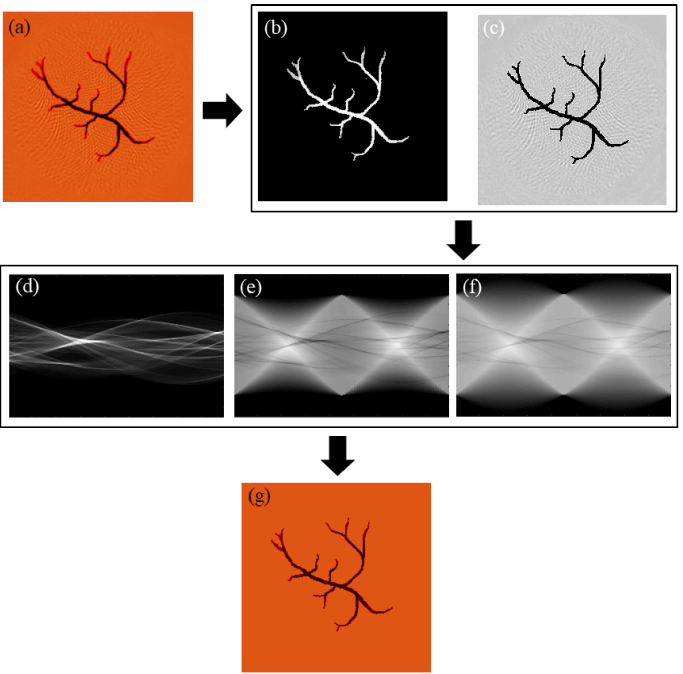
Diagram of the ABP algorithm. (a) Results of the traditional BP algorithm; (b) and (c) threshold segmentation of the target and non-target areas respectively of the image in (a); (d) and (e) Radon transformations of images (b) and (c), respectively; (f) Corrected projection area; (g) Reconstructed image of (f).

In this section, a new adaptive back-projection (ABP) algorithm is proposed to reduce the negative artifacts and the background noise in PACT images. Figure 3 shows the results of a digital phantom experiment. Figure 3(a) shows an image $I_{\Omega }(x,y)$ which was reconstructed using the BP algorithm, the negative artifacts at the image edge and the rotating blur artifacts around the image can be seen. The images $I_{\Omega_{T}}(x,y)$ and $I_{\Omega_{B}}(x,y)$ shown in Fig. 3(b) and 3(c) are obtained by adaptive threshold segmentation of the reconstructed image $I_{\Omega }(x,y)$ based on the target (the vessel-shaped feature) and non-target areas respectively. The image $I_{\Omega_{T}}(x,y)$ only retains the target area and the image $I_{\Omega_{B}}(x,y)$ only retains the background area and not the target region. In this case, the target area was the main contamination source, which caused serious artifacts in the entire image.

Radon transformation of the image $I_{\Omega_{T}}(x,y)$ can be used to obtain the boundary $\left[m_{\theta },n_{\theta }\right]$ of the target projection area $R_{\Omega_{T}}$ under each projection angle *θ* in the projection space, as shown in Fig. 3(d). Similarly, Radon transform can be performed on the image $I_{\Omega_{B}}(x,y)$ to obtain the background projection region $R_{\Omega_{B}}$ , as shown in Fig. 3(e). It can also be seen from Fig. 3(d) that the target projection is the main cause of artifacts in Fig. 3(a). The Radon transformation can be expressed as:

(2) $$R(\rho ,\theta )=\underset{D}{\int \;\;\;\int }I(x,y)\delta (xcos\theta +ysin\theta -\rho )dxdy.$$

where *D* represents the entire image plane. I(x,y) is the gray value of the pixel at the coordinate (x,y) . δ(⋅) is the Dirac delta function. ρ is the distance from the (x,y) plane straight line to the origin and θ is the angle between the normal of the straight line and the *x*-axis.

Here, we replace the background projection $R_{\Omega_{B}}(\alpha )$ with the result $R_{\Omega_{L}}(\alpha )$ after linear interpolation, which can be expressed as:

(3) $$R_{\Omega_{B}}(\alpha )\approx R_{\Omega_{L}}(\alpha )=\frac{n_{\theta }-\alpha }{n_{\theta }-m_{\theta }}R_{\Omega }(m_{\theta })+\frac{\alpha -m_{\theta }}{n_{\theta }-m_{\theta }}R_{\Omega }(n_{\theta }).$$

where $\alpha \in [m_{\theta },n_{\theta }]$ . Since the negative artifacts are mainly caused by the large projection value of an object with a high attenuation coefficient, the target projection value needs to be attenuated. For the reconstructed target image to reflect its hierarchical structure, an adaptive attenuation factor is used for the target projection. Thus, the projection $R_{\Omega_{A}}$ after attenuation and interpolation is formulated as:

(4) $$R_{\Omega_{A}}(\alpha )=K\cdot \frac{w^{\left(CT\right)}(\alpha )}{w_{max}^{\left(CT\right)}(i)}R_{\Omega_{T}}(\alpha )+R_{\Omega_{B}}(\alpha ).$$

where *K* is a constant, which is used to adjust the brightness of the whole $R_{\Omega_{T}}$ relative to $R_{\Omega_{B}}$ , that is the contrast. The smaller the amplitude of $R_{\Omega_{T}}$ , the fewer the negative artifacts produced after reconstruction. After multiple experimental validations, the contrast is optimal when the value of *K* lies between 0.2 and 0.5. $w^{\left(CT\right)}(\alpha )$ is the suppression weight used for minimizing the influences of the target source on the image. A simple but effective design of $w^{\left(CT\right)}(\alpha )$ is

(5) $$w^{\left(CT\right)}(\alpha )=tan\left\{\frac{R_{\Omega }(\alpha )}{R_{\Omega_{max}}(i)}\cdot \left(\frac{\pi }{2}-k\right)\right\}.$$

where $i\in [m_{\theta },n_{\theta }]$ , k∈(0,π/2] , and the function of *k* is to adjust the value of $R_{\Omega_{A}}(\alpha )$ after tangent function transformation to improve the contrast of the reconstructed image. The projected $R_{\Omega_{A}}$ after interpolation and correction is given in Fig. 3(f). The reconstructed image is obtained after radon inverse transformation is performed on $R_{\Omega_{A}}$ , as shown in Fig. 3(g).

The ABP algorithm can be summarized as follows:

Step 1. Rough reconstruction of initial pressure using Eq. (1).
Step 2. Extract the target area image and determine the projection area of the target object
with Eq. (2).
Step 3. Adaptive attenuation projection value with Eq. (3) and Eq. (4).
Step 4. Reconstruction of the corrected projection data.

## 3. Simulation study

**Fig. 4.**
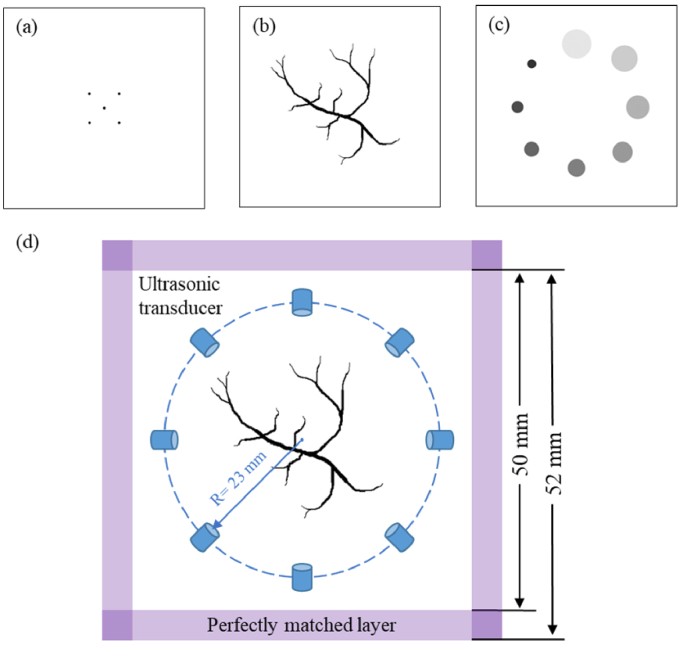
(a) Five-point source phantom (the first phantom), each with a diameter of 0.5 mm. (b) Blood vessel phantom (the second phantom). Initial pressure for the white region is equal to 0, and for the black region, it is considered as 1. (c) Eight absorbing spheres (the third phantom). From large to small, the diameter is 10 mm to 3 mm, and the interval is 1 mm; the initial pressure is 0.1 to 0.8, and the interval is 0.1. (d) Illustration of the simulation geometry.

In the simulation study, three numerical phantoms are used: the first phantom is a five-point source phantom; the second phantom is a blood vessel phantom; the third phantom is eight absorbing spheres. The first phantom consisted of five-point sources, each of which is 0.5 mm in diameter, as displayed in Fig. 4(a). This phantom allowed us to study how the axial and lateral resolutions of the point-like PA sources would depend on the UT aperture size in PACT imaging. The second phantom is vessel-shaped, as shown in Fig. 4(b). Finally, to illustrate the performance of the proposed algorithm for tissues with different absorption coefficients, the third phantom was designed to include eight spherical membranes with different degrees of absorption, as shown in Fig. 4(c). The first and second phantoms are used as binary images (the targets’ positions are denoted by 1 in the matrix with 0 elsewhere) and the third phantom is included in the gray image within the code to simulate the PA signals using the MATLAB toolbox k-Wave [27]. The corresponding computational setup is presented in Fig. 4(d). The total imaging area used in the calculation was 50 × 50 mm with a resolution of 0.1 mm. An absorption layer with a width of 2 mm was set up at the boundary of the imaging area. The phantom was placed at the center of the imaging region. The imaging area was uniformly surrounded by 360 detectors which scanned the PA signal at a radius of 23 mm. Flat detectors with diameters of 10 mm, 6 mm and 2 mm were used to investigate how the aperture size affected the PACT resolution, all with a center frequency of 2.5 MHz and a bandwidth of 75%. In addition, 40 dB of random noise was added to the collected data. The PA pressure values were recorded at 2226 time points for each position, and the sampling interval was 21 ns. The speed of sound was set at 1500 m/s and the acoustic property of the medium was homogeneous.

**Fig. 5.**
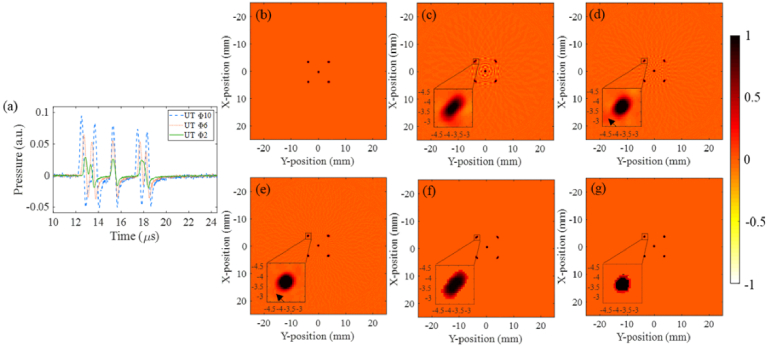
Simulation results of the first phantom, which contains five-point sources. (a) Comparison of the PA signals recorded by the UTs with aperture diameters of 10 mm, 6 mm and 2 mm; (b) Initial pressure of five-point sources phantom, each with a diameter of 0.5 mm; (c)-(e) Reconstructed images obtained using the BP algorithm for the UTs with aperture diameters of 10 mm, 6 mm and 2 mm, respectively; (f) Reconstructed image using the ABP algorithm for the UT with 10 mm aperture; (g) Reconstructed image using the CODA method. The insets in (c)-(g) show magnified views of the point source in the dotted box.

Figure 5 presents the reconstructed results of the five-point sources with the conventional FBP algorithm and our proposed method. Here, Fig. 5 (a) shows the PA signals recorded by the UTs with aperture diameters of 10 mm, 6 mm and 2 mm, respectively. It can be seen that as the aperture size decreases, the duration of PA signals is shortened, which reduces the mixing of useless signals. However, the amplitude of the signals is also greatly attenuated. Figures 5(c)–5(e) are obtained by the traditional BP algorithm using UTs with aperture diameters of 10 mm, 6 mm and 2 mm, respectively. It may be noted that the reconstruction of all three images is ideal when the source is located at the scanning center, while point sources situated away from the center have elongated lateral shapes, that is, the spinning artifacts. Furthermore, the elongation increases as the aperture size of the UT increases. Figure 5(e) illustrates that the lateral elongation of the surrounding point sources is reduced significantly when compared with Fig. 5(c) and 5(d). This means that using UTs with a small aperture size can effectively improve the lateral resolution of the image. Nevertheless, we also noticed negative artifacts [indicated by the black arrows in Fig. 5(d)–5(e)] and background noise that remains in the reconstructed image. When the proposed ABP algorithm is directly used for the UT with 10 mm aperture, although artifacts and noise can be eliminated, rotation blur still exists, as shown in Fig. 5(f). Figure 5(g) illustrates the reconstructed results using the proposed CODA method (applying the ABP algorithm to the data shown in Fig. 5(e)). It can be seen that the negative artifacts and background noise in the image are almost eliminated and the spinning artifacts are reduced as well.

**Fig. 6.**
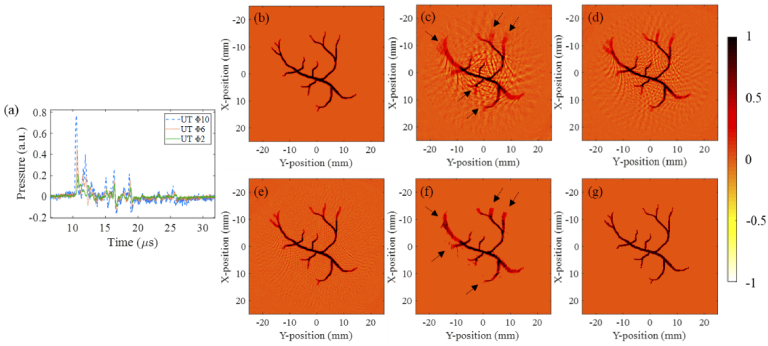
Simulation images of the second phantom. (a) Comparison of the PA signals recorded by the UTs with aperture diameters of 10 mm, 6 mm and 2 mm; (b) Initial pressure of the vessel-shaped phantom; (c)-(e) Reconstructed images obtained by the BP algorithm using the UTs with aperture diameters of 10 mm, 6 mm and 2 mm, respectively; (f) Reconstructed image using the ABP algorithm for the UT with 10 mm aperture; (g) Reconstructed image using the CODA method.

Normalized reconstruction images of the vessel-shaped phantom are shown in Fig. 6. Here, Fig. 6(a) shows the PA signals recorded by the UTs with aperture diameters of 10 mm, 6 mm and 2 mm, respectively. Figures 6(c)–6(e) are obtained by the traditional BP algorithm using flat UTs with aperture diameters of 10 mm, 6 mm and 2 mm, respectively. In Fig. 6(c), target away from the imaging center that is obtained with the largest aperture size transducer are elongated in the lateral direction (that is, the spinning artifacts), as indicated by the black dashed arrows. Similar observations can be drawn from Fig. 6(c)–6(e). These distortions are greatly reduced as the transducer aperture size decreases. Again, we also noticed in Fig. 6(e) that the reconstructed image still contains some background noise and negative artifacts, even for the smallest aperture size transducer. In addition, the image intensity of the vessel tail is greatly attenuated. Using the proposed ABP algorithm directly for the UT with 10 mm aperture can eliminate noise, but rotation blur still exists [indicated by the black arrows in Fig. 6(f)], as shown in Fig. 6 (f). Figure 6(g) illustrates the reconstructed results using our proposed CODA method (applying the ABP algorithm to the data shown in Fig. 6(e)). Not only are the spinning artifacts in the image essentially eliminated, but the negative artifacts and background noise are also suppressed.

**Fig. 7.**
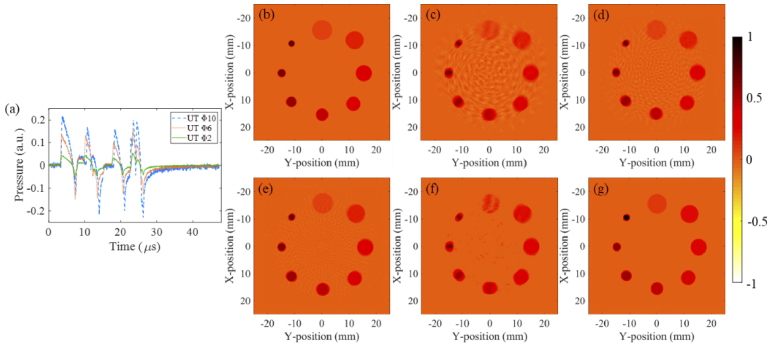
Simulation images of the third phantom, which consists of eight absorbing spheres. (a) Comparison of the PA signals recorded by the UTs with aperture diameters of 10 mm, 6 mm and 2 mm; (b) The absorbing spheres phantoms; (c)-(e) Reconstructed images obtained by the BP algorithm using the UTs with aperture diameters of 10 mm, 6 mm and 2 mm, respectively; (f) Reconstructed image using the ABP algorithm for the UT with 10 mm aperture; (g) Reconstructed image using the CODA method.

The normalized reconstruction results of eight spheres with different absorption coefficients are shown in Fig. 7, and the observations are similar to those in Fig. 5 and Fig. 6. The traditional BP algorithm works best when the UT aperture is the smallest, as shown in Fig. 7(e). However, background noise and edge artifacts still exist. In contrast, the proposed method can effectively eliminate artifacts and accurately recover images, as illustrated in Fig. 7(g). This indicates that the proposed method also has good working performance for substances with different absorption coefficients.

Two indexes for quantitative evaluation are used as the metrics to evaluate the performance of different methods, as shown in Table 1. The Peak Signal-to-Noise Ratio (PSNR) which is a conventional metric of the image quality, and the Structural Similarity Index (SSIM), a higher value indicates a better quality of the estimated image. Obviously, the smaller the UT aperture, the better the image quality. The PSNR and SSIM values of the image reconstructed by the CODA method have reached the maximum. In addition, through the quantitative evaluation of the three models, the reconstruction performance of the proposed method is more prominent in vascular tissue.

**Table 1.** Quantitative Evaluation of Different Methods in Simulation Experiment

Imaging mode Phantom	10mm-BP		6mm-BP		2mm-BP		10mm-ABP		CODA	
	PSNR	SSIM	PSNR	SSIM	PSNR	SSIM	PSNR	SSIM	PSNR	SSIM
Pencil leads	29.283	0.964	32.719	0.975	36.792	0.986	33.246	0.964	**39** **.** **124**	**0**.**994**
Blood vessel	21.993	0.647	23.423	0.716	24.751	0.799	24.542	0.845	**27**.**585**	**0**.**967**
Absorbing spheres	22.621	0.906	24.119	0.947	24.590	0.957	23.884	0.914	**31**.**362**	**0**.**990**

## 4. Experimental study

**Fig. 8.**
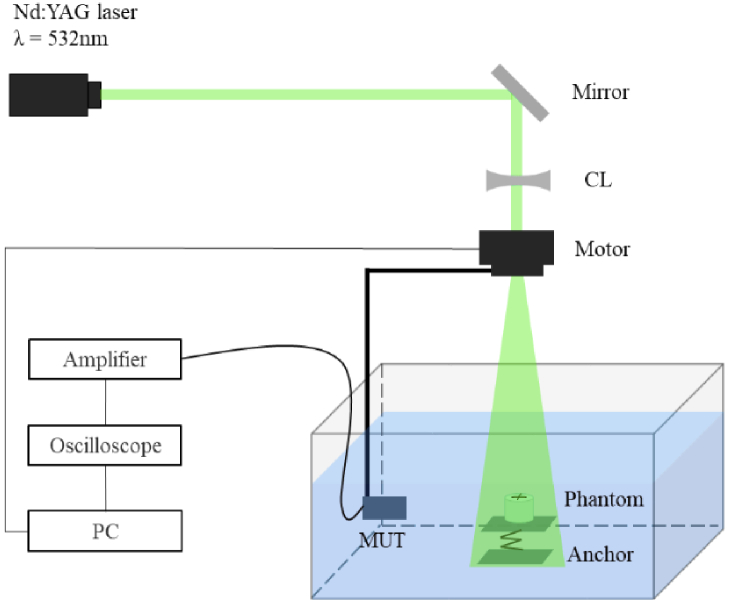
Diagram of a circular-scanning PACT system. CL, concave lens; MUT, modified ultrasonic transducer; PC, personal computer.

To further evaluate the performance of our proposed method, we conducted experiments on agar phantom and *ex-vivo* chicken heart. Both experiments were carried out successively with a conventional 2D circular scanning PACT system, as depicted in Fig. 8. A Q-switched, frequency-doubled Nd:YAG pulsed laser (TINY-200L, Grace) with a wavelength of 532 nm and 10 Hz repetition rate, was employed to excite photoacoustic waves. The laser beam was diverged by a planar-concave lens, to give a fluence of 4 mJ/cm^2^ on the phantoms’ surface. During the imaging process, the experimental objects were immersed in a water tank, and the modified ultrasonic transducer (MUT) was mounted on an electric rotating platform (TBR100, Zolix) to detect the PA signals. The receiving surface of the MUT was about 35 mm away from the rotation center. The platform was rotated through 360 steps with a step size of 1°. The PA signal was amplified by a 26 dB low-noise pre-amplifier (ZFL-500LN, Mini-Circuits) before being transmitted to an oscilloscope (MSO46, Tektronix). The oscilloscope averaged the collected PA signals and sent them to a computer for imaging processing.

### 4.1 Phantom experiments

The phantom experiment is divided into two parts from simple to complex. The first of the phantoms contained 5 pencil leads (0.5 mm diameter) which acted as point targets and the second phantom contained a complex geometry made up of multiple hairs. We embedded the two phantoms independently in two agar cylinders which were made by mixing 2 g agarose with 100 g deionized water. The water temperature was kept at 24°C for the duration of the experiments.

**Fig. 9.**
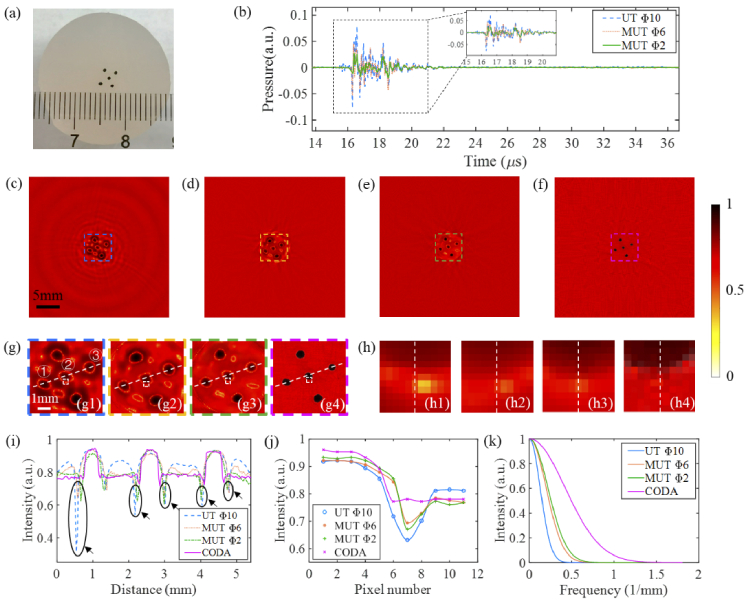
Experimental results of imaging pencil leads in agar phantom. (a) Cross-sectional photograph image of the phantom; (b) Comparison of PA signals recorded by traditional UT with an aperture of 10 mm and MUT with aperture diameters of 6 mm and 2 mm; (c)-(e) Reconstructed PACT images obtained by the BP algorithm using transducers with aperture diameters of 10 mm, 6 mm and 2 mm, respectively; (f) Reconstructed image using the CODA method; (g1)-(g4) zoomed-in views of the regions in colored dashed boxes in (c)-(f), respectively; (h1)-(h4) zoomed-in views of the regions in white dashed boxes in (g1)-(g4), respectively; (i) Comparison of the profiles along the dotted white lines in (g1)-(g4); (j) and (k) are the comparison of the intensity curves and the MTF curves along the dotted white lines in (h1)-(h4), respectively.

Figure 9 displays the reconstructed results of the first agar phantom experiment. A cross-sectional photograph image of the pencil lead phantom is shown in Fig. 9(a). Figure 9(b) shows the comparison of PA signals recorded by traditional UT with an aperture 10 mm and MUT with aperture diameters of 6 mm and 2 mm, respectively. From the time domain, it was found that the PA signal collected by UT with an aperture diameter of 10 mm is longer, indicating that it contains invalid information, which will result in spinning blurring in the reconstructed image. In contrast, the MUT collects a shorter PA signal profile. It is worth noting that compared with the simulation experiment, the amplitude attenuation of the signal acquired by the MUT is smaller. Figures 9(c)–9(e) show the reconstructed images using the conventional BP algorithm, in which Fig. 9(c) used the as-supplied UT with an aperture diameter of 10 mm, while Fig. 9(d) and 9(e) used the MUTs with aperture diameters of 6 mm and 2 mm, respectively. Elongation of point sources in the lateral direction can be observed in the reconstructed images of the point source phantom experiment. Note that the point sources can be resolved clearly using the MUT with the 2 mm aperture even at locations far from the scanning center, as shown in Fig. 9(e). However, the background noise and the negative artifacts at the edges still stubbornly exist in the reconstructed image. Figure 9(f) illustrates the reconstruction results using our proposed CODA method. The four images in Fig. 9(g) show enlarged views of the colored dotted boxes in Fig. 9(c)–9(f), from left to right respectively. It can be seen in Fig. 9(f) and 9(g4) that the reconstruction result of the CODA method is effective in eliminating the image background noise and negative artifacts.

For a quantitative comparison of the four reconstructed images, Fig. 9(i) extracts the profiles of three representative targets [numbered 1 to 3 in Fig. 9(g1)] along the four dashed white lines in Fig. 9(g1)–9(g4). It is worth noting that each of the dashed white lines covers three targets, but the blue dashed line profile for the 10 mm aperture UT shows seven arch-shaped peaks while the dotted orange line (6 mm aperture) shows five. This is because large aperture UTs limit the receiving angle of PA signals, resulting in distortion of the image and interference patterns. In contrast, the green dotted line shows three arched peaks, accurately reflecting the three targets. This is because the MUT with an aperture diameter of 2 mm can receive PA signals over a large acceptance angle. Nevertheless, the bottoms of the arches are too low for the 10 mm, 6 mm and 2 mm aperture curves [as indicated with black arrows in Fig. 9(i)] reflecting the fact that negative artifacts appear at the edges of the image in the reconstruction results. On the other hand, the purple curve clearly shows the three arches and the curve between the arches is flat. In addition, the width of each arch is narrower than those of the blue curve. This result not only confirms that the CODA method eliminates the negative artifacts and further improves the lateral resolution of the image, but also performs well for objects placed at different distances from the UT.

To quantitatively compare the performance of the different imaging modes, we captured four areas measuring 11×11 pixels from the edge of the image. These areas are indicated by the white dotted boxes in Fig. 9(g1)–9(g4), and the corresponding 11×11 pixel images are shown in Fig. 9(h1)–9(h4). Figures 9(j)–9(k) show the intensity curves and modulation transfer function (MTF) curves corresponding to the white dotted lines in Fig. 9(h1)–9(h4) respectively. As seen in Fig. 9(j), the curves corresponding to the 6 mm aperture (orange) and 2 mm aperture (green) have steeper edges than that of the 10 mm aperture detector (blue). However, each of these three intensity curves has a “V” shape and the position of the target edge is not clear. Meanwhile, the CODA curve (purple) is flatter at the bottom, and the edge of the target is easy to identify at the sixth pixel. This indicates the ability of the CODA method to remove negative artifacts. As shown in Fig. 9(k), the MTF curve of the CODA (purple) curve is much wider than those of the other three MTF curves. This confirms that the CODA method improves the lateral spatial resolution compared with the conventional UT method when using an aperture of 10 mm.

**Fig. 10.**
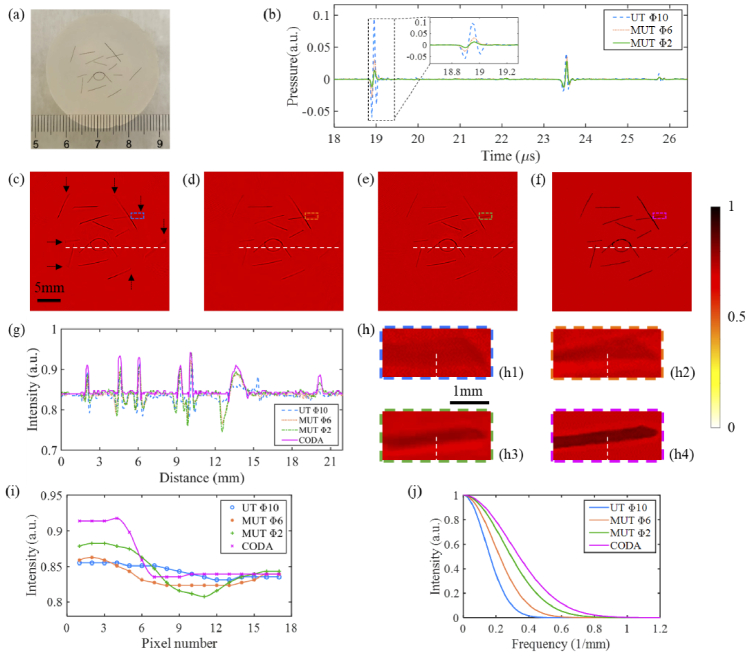
Experimental results of imaging the complex geometry of agar phantom. (a) Cross-sectional photograph image of the phantom; (b) Comparison of PA signals recorded by traditional UT with an aperture of 10 mm and MUT with aperture diameters of 6 mm and 2 mm; (c)-(e)Reconstructed PACT images obtained by the BP algorithm using transducers with aperture diameters of 10 mm, 6 mm and 2 mm, respectively; (f) Reconstructed image using the CODA method; (g) Comparison of the profiles along the dotted white lines in (c)-(f); (h1)-(h4) zoomed-in views of the regions in colored dashed boxes in (c)-(f), respectively; (i) and (j) are the comparison of the intensity curves and the MTF curves along the dotted white lines in (h1)-(h4), respectively.

To demonstrate the feasibility of applying the CODA method to imaging objects with different geometric shapes, we used an irregular geometry phantom that was made up of human hairs. The reconstructed image results are shown in Fig. 10. The comparison of PA signals recorded by traditional UT and the MUT is shown in Fig. 10(b). The PA signal collected by the MUT is seen to be shorter than that by the traditional UT with a large size in the time domain. This means that the spinning blur of the reconstructed image can be eliminated by using the MUT. It can be seen that the tail of the hair is widened in the lateral direction [shown clearly by the black arrows in Fig. 10(c)] when using a traditional UT, as shown in Fig. 10(c). While these spinning blurs are relieved when using the smaller aperture MUTs, as shown in Fig. 10(d) and 10(e). Likewise, there are negative artifacts at the edge of the target. While the unnecessary artifacts are almost eliminated by the CODA method, as demonstrated in Fig. 10(f). Figure 10(g) shows a comparison of the profiles along the dotted white lines in Fig. 10(c)–10(f). Compared with the blue curve, the peak of the purple curve is higher and narrower, and the trough is very small, which successfully indicates the superiority of the CODA method in PACT again.

To reflect the improvement of the CODA method more clearly, part of the reconstruction results is intercepted and enlarged, as shown in Fig. 10(h1)–10(h4). Figures 10(i)–10(j) show the intensity curves and MTF curves corresponding to the white dotted lines (through 17 pixels) in Fig. 10(h1)–10(h4), respectively. As can be seen from Fig. 10(i) and 10(j), the CODA (purple) curve has a steeper edge and its MTF curve is wider. This again confirms that the CODA method also improves the lateral spatial resolution and eliminates negative artifacts when imaging line targets. This suggests the CODA technique would be highly effective in clinical applications such as imaging blood vessels.

### 4.2 Ex-vivo experiments

**Fig. 11.**
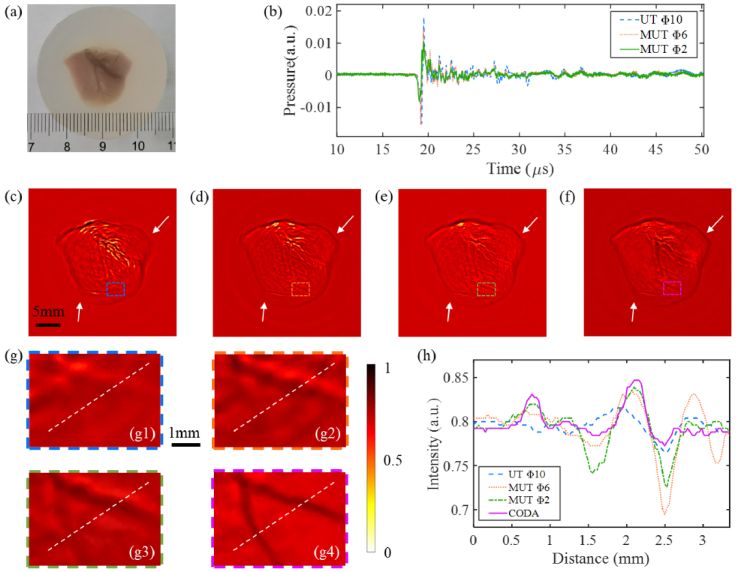
Experimental results of imaging *ex-vivo* chicken heart. (a) Cross-sectional photograph image of the phantom; (b) Comparison of PA signals recorded by traditional UT with an aperture of 10 mm and MUT with aperture diameters of 6 mm and 2 mm; (c)-(e) Reconstructed PACT images obtained by the BP algorithm using transducers with aperture diameters of 10 mm, 6 mm and 2 mm, respectively; (f) Reconstructed image using the CODA method; (g1)-(g4) zoomed-in views of the regions in colored dashed boxes in (c)-(f), respectively; (h) is the comparison of the profiles along the dotted white lines in (g1)-(g4).

To further validate the performance of the CODA method for biological tissue applications, isolated chick hearts were used for *ex-vivo* experiments. Similarly, a chicken heart was embedded into a cylinder of agar at a concentration of 2%, as shown in Fig. 11(a). Figure 11(b) shows the comparison of PA signals recorded by traditional UT with an aperture of diameters of 10 mm and the MUT with aperture diameters of 6 mm and 2 mm, respectively. Figures 11(c)–11(e) show the reconstructed images using the conventional BP algorithm, in which Fig. 11(c) used the as-supplied UT with an aperture diameter of 10 mm, while Fig. 11(d) and 11(e) used the MUT with aperture diameters of 6 mm and 2 mm, respectively. Figure 11(f) illustrates the reconstructed image of the CODA method. By comparing the images reconstructed with different methods, we found that the CODA method provided the best edge contour [shown by the white arrows in Fig. 11(c)–11(f)] and displayed the vessels most clearly. Figures 11(g1)–11(g4) show enlarged views of the colored dotted boxes in Fig. 11(c)–11(f), respectively. The vessels are seen most clearly, with the thinnest diameter in Fig. 11(g4).

Figure 11(h) shows a comparison of the profiles along the dotted white lines in Fig. 11(g), which again indicates the CODA method improves the resolution and removes the negative artifacts in PACT. These results successfully validated that our method works well for biological tissue PA imaging.

## 5. Discussion and conclusion

In this work, we proposed and demonstrated a new CODA method for reconstructing PACT images by removing imaging artifacts that are caused by the limited angular field of view of UTs. In addition to the proposed CODA method, several hardware modifications have been proposed to improve the lateral resolution, such as using the small diameter, high numerical aperture transducers and adding negative acoustic lenses. However, these have inherent limitations such as low sensitivity, and high cost and can be complicated to implement. Our strategy is to employ a damping device with a controllable active area on the receiving surface of a large-aperture UT without changing the area of the piezoelectric crystal inside the UT. This means that the sensitivity of the detector is less impacted by the small aperture size, and the detectors can be readily incorporated into most current PACT systems, which would be a straightforward and low-cost modification. However, the performance comparison between this modified UT and the actual small aperture UT needs further study. Compared with deep learning or model-based reconstruction methods, the proposed ABP algorithm is a back-projection-based method, free of time-consuming data training and does not require large computing resources and expensive hardware.

The accuracy of extracting the signal associated with the sample target relies on the selection of an appropriate threshold. Inappropriate selection of the threshold will result in excessive noise and artifacts or a very weak noisy signal. Our proposed segmentation method is an adaptive threshold method based on the global image. If the absorption coefficient of the target tissues is heterogenous, simple thresholding is not effective in practical clinical diagnosis. In the future, we aim to study more accurate locally-based adaptive multi-threshold segmentation methods. Finally, the application of our proposed method to *in-vivo* biomedical imaging has not been tested in this work and needs to be studied soon.

Here we report for the first time the application of the CODA method to improve the lateral spatial resolution in PACT. Both simulations and experimental results demonstrate that this method can significantly improve the lateral resolution in PACT images. In addition, the proposed ABP algorithm can effectively suppress the background noise and unwanted artifacts at the edges of the imaged targets. Experiments on phantom and *ex-vivo* samples show the effectiveness of the method. A single-element circular scanning system constructed using this method can offer the same performance as traditional 2D PACT systems with a large array of detectors, greatly reducing the cost and system complexity.

## Data Availability

Data underlying the results presented in this paper are not publicly available at this time but may be obtained from the authors upon reasonable request.
